# Whole genome sequencing in ROHHAD trios proved inconclusive: what’s beyond?

**DOI:** 10.3389/fgene.2023.1031074

**Published:** 2023-08-07

**Authors:** A. Grossi, M. Rusmini, R. Cusano, M. Massidda, G. Santamaria, F. Napoli, A. Angelelli, D. Fava, P. Uva, I. Ceccherini, M. Maghnie

**Affiliations:** ^1^ Laboratory of Genetics and Genomics of Rare Diseases, IRCCS Istituto Giannina Gaslini, Genova, Italy; ^2^ Clinical Bioinformatics, IRCCS Istituto Giannina Gaslini, Genova, Italy; ^3^ CRS4, Science and Technology Park Polaris, Pula, Italy; ^4^ Pediatric Clinic and Endocrinology, IRCCS Istituto Giannina Gaslini, Genova, Italy; ^5^ D.I.N.O.G.M.I, Università degli Studi di Genova, Genova, Italy

**Keywords:** ROHHAD, WGS, rare disease, pediatric disorder, hypoventilation, hypothalamic dysfunction

## Abstract

Rapid-onset Obesity with Hypothalamic dysfunction, Hypoventilation and Autonomic Dysregulation (ROHHAD) is a rare, life-threatening, pediatric disorder of unknown etiology, whose diagnosis is made difficult by poor knowledge of clinical manifestation, and lack of any confirmatory tests. Children with ROHHAD usually present with rapid onset weight gain which may be followed, over months or years, by hypothalamic dysfunction, hypoventilation, autonomic dysfunction, including impaired bowel motility, and tumors of neural crest origin. Despite the lack of evidence of inheritance in ROHHAD, several studies have been conducted in recent years that have explored possible genetic origins, with unsuccessful results. In order to broaden the search for possible genetic risk factors, an attempt was made to analyse the non-coding variants in two trios (proband with parents), recruited in the Gaslini Children’s Hospital in Genoa (Italy). Both patients were females, with a typical history of ROHHAD. Gene variants (single nucleotide variants, short insertions/deletions, splice variants or in tandem expansion of homopolymeric tracts) or altered genomic regions (copy number variations or structural variants) shared between the two probands were searched. Currently, we have not found any potentially pathogenic changes, consistent with the ROHHAD clinical phenotype, and involving genes, regions or pathways shared between the two trios. To definitively rule out the genetic etiology, third-generation sequencing technologies (e.g., long-reads sequencing, optical mapping) should be applied, as well as other pathways, including those associated with immunological and autoimmune disorders, should be explored, making use not only of genomics but also of different -omic datasets.

## Introduction

Rapid-onset Obesity with Hypothalamic dysfunction, Hypoventilation and Autonomic Dysregulation (ROHHAD) is a rare pediatric disorder of unknown etiology, characterized by increased morbidity and mortality of up to 50%–60% from hypoventilation and cardiopulmonary arrest (Ize-Ludlow et al., 2007; Lazea et al., 2021). To date, fewer than 200 cases in the world are known from the literature (Ceccherini et al., 2022). The first manifestation of this syndrome is almost always rapid-onset obesity, which usually occurs between 2 and 4 years of age in a previously healthy child. Over the following years, with variable timing, one or more signs of hypothalamic dysfunction appear: hyperprolactinemia, growth hormone deficiency, central hypothyroidism, central adrenal insufficiency or Cushing syndrome, early or delayed puberty, water-electrolyte balance disorders. Dysautonomia signs, such as—but not limited to—heart rhythm anomalies, excessive sweating, gut dysmotility, have been reported at a median age of 4.95 years, and central hypoventilation at 5.33 years (Harvengt et al., 2020). Hypoventilation is often preceded by obstructive sleep apnea syndrome (Reppucci et al., 2016). A neural tumor—ganglioneuroma, ganglioneuroblastoma or neuroblastoma - has been reported in approximately half of the patients, and most of these tumors are diagnosed less than 2 years after initial weight gain. The diagnosis of ROHHAD syndrome is not always straightforward, and the risk of misdiagnosis is elevated, especially at disease onset, since clinical manifestations—especially the fatal ones—may appear over time (Reppucci et al., 2016) therefore, early identification of patients and early treatment of clinical manifestations is crucial for patient survival. Management of this severe condition includes hormonal replacement therapy, non-invasive or invasive ventilation, lifestyle interventions such as hypocaloric diet and physical activity, treatment of further comorbidities such as diabetes and dyslipidemia, and neural tumor resection in some cases (Lazea et al., 2021; Ceccherini et al., 2022; Tiwari et al., 2022). Based on the evidence of immunological dysfunction, immunosuppressive or immunomodulatory therapies—such as intravenous immunoglobulins, steroids, cyclophosphamide, rituximab, mycophenolate and plasmapheresis—have been attempted in isolated cases, with partial improvement of some clinical manifestations (Chow et al., 2015; Giacomozzi et al., 2016; Jacobson et al., 2016; Hawton et al., 2022). Improvement after immunomodulatory or immunosuppressive therapy was only transient in some cases. Up to now, no therapeutic protocol is available for ROHHAD syndrome, and patient management remains challenging. The underlying aetiology is still unknown and several hypotheses have been formulated, of which the genetic one is among the most accredited and explored so far, also given the clinical proximity to Congenital Central Hypoventilation Syndrome (CCHS) and Prader-Willi syndrome (PWS) (Patwari et al., 2011; Rand et al., 2011; Barclay et al., 2015; Chow et al., 2015; Thanker et al., 2015; Barclay et al., 2016; Barclay et al., 2018; Lee et al., 2018; Calvo et al., 2019; Giacomozzi et al., 2019; Lazea et al., 2021; Artamonova et al., 2022; Ceccherini et al., 2022; Mandel-Brehm et al., 2022). However, the genetic etiology of ROHHAD lacks evidence such as intra-family segregation, and also the failure to detect single gene defects allows to exclude a simple mendelian inheritance. In fact, Barclay et al. (2015) studied 35 subjects through Whole Exome Sequencing (WES) without identifying disease variants or associated genes and other groups have also focused on possible candidate genes without any success. In particular, the genes responsible for the development and function of the hypothalamic and the neuroendocrine and autonomic systems, such as *HCRT*, *HCRTR1*, *HCRTR2*, *MAGEL2*, *NDN*, *OTP*, and *PACAP*, have thoroughly been investigated but with unsuccessful results (Patwari et al., 2011; Rand et al., 2011; Barclay et al., 2015; Thanker et al., 2015; Barclay et al., 2016).

Lack of results using such a genomic approach does not rule out an oligogenic or multifactorial mode of inheritance in ROHHAD, nor the presence of important structural variants or copy number variations, variants occurring outside the coding regions, splice defects or trinucleotide repeat expansions. Therefore, in order to broaden the search for genetic risk factors, we decided to perform Whole Genome Sequencing (WGS) in two trios (proband with parents) selected from a set of 23 ROHHAD patients, recruited so far in the Pediatric Endocrinology of the Gaslini Children’s Hospital in Genoa, Italy. The working hypothesis involves the search for genes or genomic regions shared between the two probands, and the selection of those genetic factors potentially responsible for a severe clinical phenotype resembling ROHHAD.

## Methods

**FIGURE 1:**
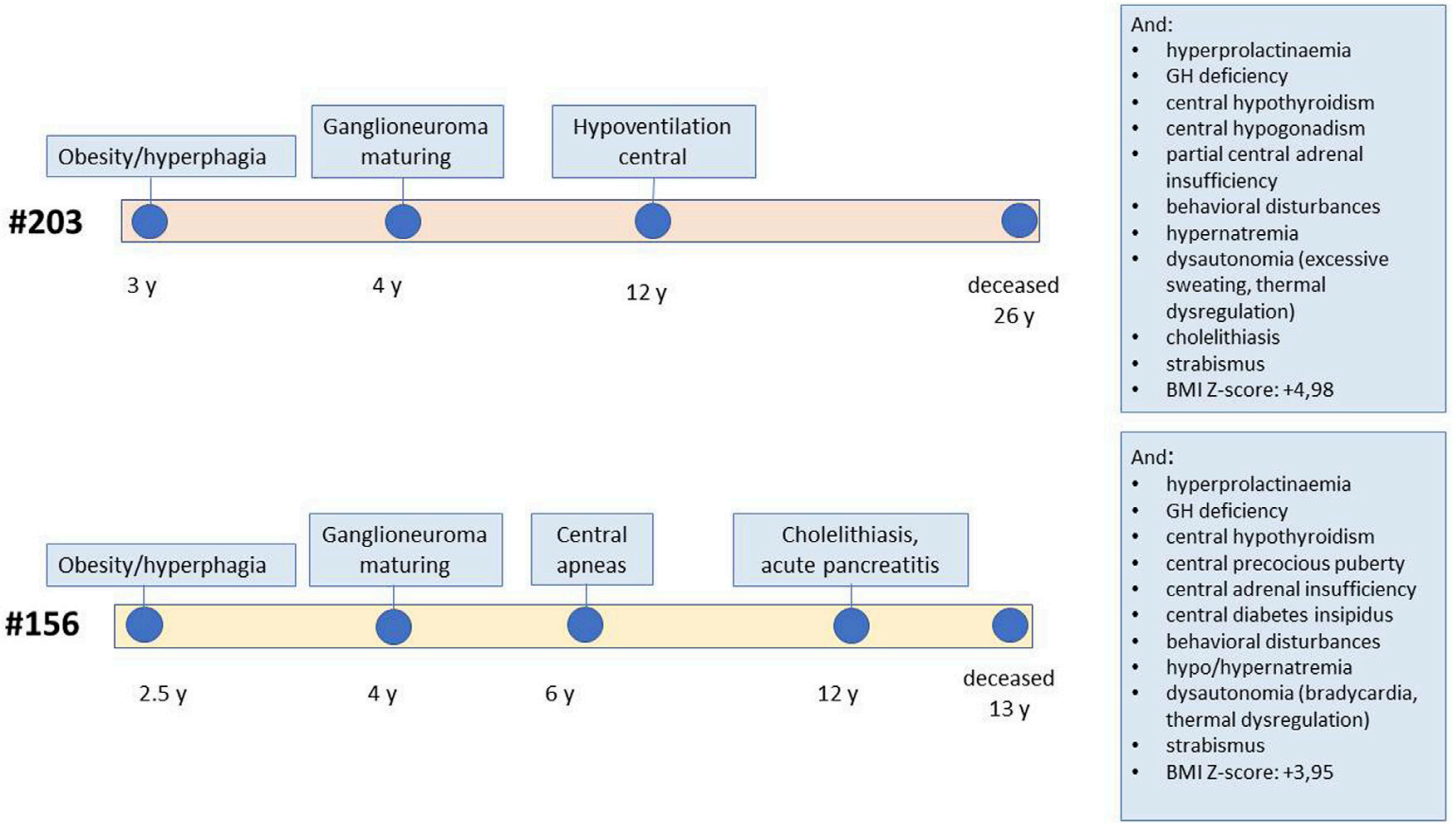
Schematic representation of the clinical progression in the two patients. The figure reports the age of onset of specific ROHHAD symptoms for each of the two study patients, from first manifestation to death. The boxes report additional symptoms.

The two patients selected for genetic analysis were females with a history of rapid-onset obesity in early childhood, abdominal ganglioneuroma that was confirmed by pathology, sleep-disordered breathing requiring non-invasive ventilation, and multiple pituitary hormone defects developed over time (patient #156 showed partial central diabetes insipidus, central precocious puberty, growth hormone deficiency, central hypothyroidism and central adrenal insufficiency, while patient #203 was diagnosed with central hypogonadism, growth hormone deficiency, central hypothyroidism and partial central adrenal insufficiency), asymptomatic hyperprolactinemia, and behavioural symptoms (Figure 1; Supplementary Material S1). These were selected, among all patients followed at our centre, as they carried all the signs and symptoms of severe ROHHAD, with a manifestation and progression of the clinical phenotype very similar between the two patients. Both patients’ brain MRIs were normal except for partial empty sella in patient #203 and temporal lobe arachnoid cyst in patient #156. They both died from cardiorespiratory failure: patient #203 at age 25, patient #156 at age 12. Standard karyotype, CGH array, methylation test for Prader-Willi syndrome were normal in both patients.

The six DNA samples belonging to the two trios’ members were extracted from peripheral blood by using QIAamp DNA Blood Midi kit (Qiagen, Germantown, MD, USA). The quality and quantity of DNA thus obtained were determined by the Nanodrop. The two probands’ DNA samples were previously tested and confirmed negative for *PHOX2B* mutations by Sanger sequencing.

Patient’s genomic DNA Sequencing library for Whole Genome Sequencing was prepared according to manufacturer instructions for the Nextera DNA Library Prep kit (Illumina). Quality of libraries was assessed using DNA1000 Chip on the BioAnalyzer 2,100 (Agilent) and Qubit fluorimetric quantitation using Qubit dsDNA BR Assay Kits (Invitrogen). Libraries were loaded into Paired Ends flow cells on an Illumina cBot followed by indexed 150 bp paired-end sequencing on a HiSeq 3,000 using SBS Kit (Illumina). We achieved an average coverage of 30 ×.

The raw data was first analysed with FASTQC tool (Andrews S. (2010) for quality control, reports were integrated by MultiQC v1.10 (Ewels et al., 2016) and then FASTQ were processed for variant detection both in the coding and non-codingregions, using the genome assembly GRCh38 (hg38) as reference (Supplementary Figure S1).

For FASTQ data processing, the commercial Illumina DRAGEN (Dynamic Read Analysis for GENomics) Bio-IT Platform, based on GATK Best Practices (Van der Auwera GA and O’Connor BD, 2020), was used. In particular, the Germline Pipeline was applied to all single samples, while the Joint Genotyping Pipeline (v.3.8.4) was used to combine data from each trio and to identify single nucleotide variants (SNVs) and Indels, Copy Number Variations (CNVs), Structural Variants (SVs), and repeat expansions.

According to an independent evaluation performed on real and synthetic datasets (Zhao et al., 2020), F1-score, recall and precision values of DRAGEN (without Variant Quality Score Recalibration post-processing as in this study) for SNPs calling were 0.99 in all three metrics. Metrics for Indel calls were more diverse with F1-score, recall and precision higher than 0.93, 0.95 and 0.91, respectively. It is worth mentioning that metrics for the real dataset were based on truth callset which excludes more difficult regions to sequence and analyze (e.g., repeats and segmental duplications) as consensus in such regions has not been reached. For the synthetic dataset, the truth callset was based on the PacBio technology and should enable benchmarking in regions that are difficult to map with short reads. However, it may include errors that were intrinsically present in the long reads. These performance metrics, specifically in hard-to-sequence regions, are probably not as high as those reported by Zhao et al., and therefore we may have a lower accuracy in the detection of variants in these regions.

DRAGEN relies on Manta (Chen et al., 2016) for SV analysis. In a recent comparison of 14 SV analysis tools (Cameron et al., 2019), Manta was selected among the best-performing caller, with F1-score, recall and precision metrics of 0.70, 0.88 and 0.59, respectively.

Detection of CNVs is based on a depth-based approach implemented in Illumina Canvas software (Roller et al., 2016). According to Canvas’ developers, WGS germline CNV calling metrics for accuracy, precision and recall were 0.79, 0.96 and 0.78 respectively.

DRAGEN also includes ExpansionHunter (Dolzhenko et al., 2019) to accurately call repeat expansions using sequence graphs. The presence/absence of a set of clinically relevant repeat expansions, curated by the DRAGEN team, is provided as output by the software. Based on a benchmark from the software’s developers, ExpansionHunter achieved a precision of 0.91 and a recall of 0.99 in detecting the expanded repeats from short-reads WGS with experimentally confirmed repeat expansions.

*De novo* variants were identified using both Dragen and DeNovoGear (Ramu et al., 2013), a software for detection of *de novo* mutations available in the trio-dnm2 plugin of bcftools (v.1.11). DeNovoGear results were filtered using a posterior probability cutoff of 0.5. This threshold corresponds to a sensitivity equal to 1 and a False Discovery Rate equal to 0.8283 (Ramu et al., 2013). We decided to use a less stringent threshold to call *de novo* variants on the two probands and then select *de novo* variants present in both patients.

The VCF files were also used to exclude consanguinity within each parental couple and to confirm familial-relationships and gender of all the individuals (Pedersen and Quinlan, 2017).

All SNVs and indels variants were annotated with VEP (Variant Effect Predictor) (v.100.2) (McLaren et al., 2016). The annotation included the allele frequencies and the functional gene constraints (gnomAD) (Karczewski, 2020), several pathogenicity predictions (e.g., CADD, DANN, SIFT, Polyphen) (Adzhubei et al., 2013; Quang et al., 2015; Vaser et al., 2016; Rentzsch et al., 2018) and the conservation score (GERP) (Davydov et al., 2010). Moreover, variants were annotated with ClinVar database (https://www.ncbi.nlm.nih.gov/clinvar/) and the splicing predictions provided by SpliceAI v.1.3 (Jaganathan et al., 2019). For SpliceAI, we focused on the variants with a score > 0.5 in at least one of the four predictions (acceptor/donor gain, acceptor/donor loss). Due to the rarity of the disease, only variants with allele frequency less than 0.001 in the healthy population were further considered, taking into account all possible segregation models (*de novo* and recessive inheritance, including compound heterozygosity) using Slivar (v.0.1.12) (Pedersen et al., 2021).

Coding and non-coding variants were also considered separately. To predict the pathogenicity of structural variants, the VCF files were annotated by AnnotSV v.3.1.1 (Geoffroy et al., 2021). The Integrative Genome Viewer (IGV) Robinson et al. (2011) was used for the visual exploration of genomic data. The Database of Genomic Variants (DGV) (http://dgv.tcag.ca/dgv/app/home) was interrogated to filter known benign structural variants.

For targeted NGS re-sequencing of the *SERPINE2* gene, about 50 Kbp of the genomic region, spanning 65 Kbp from 5′UTR to 3′UTR, was amplified with the exclusion of a portion of intron 1. For each of the additional 22 samples, equimolar pools of the resulting five PCR products, partially overlapping and with a size range between 7.4 Kbp and 16.5 Kbp, were processed with the Nextera XT (Illumina) library preparation kit. Indexed libraries were sequenced on a MiSeq instrument (Illumina) with MiSeq v3-600 cycle cartridge to obtain 200X average coverage.

## Results

At first, only the #156 trio underwent WGS, revealing a *de novo* splicing variant in the *SERPINE2* gene that altered the transcription product. The expression of SERPINE2 in the brain and its role as a modifier in neurogenesis led us to hypothesize a possible role also in the pathogenesis of ROHHAD (Monard D., 2017). We therefore decided to screen the whole genomic portion of the *SERPINE2* gene by Sanger sequencing and Long-PCR targeted NGS re-sequencing in 22 additional ROHHAD or suspected-ROHHAD patients, made available in the meantime, without finding any pathogenic variant (data not show). Results confirmed the *de novo* splicing variant in sample #156 and other known SNPs but no additional variants deserving further investigations were detected in the sample cohort.

Successively, also the second trio (#203) underwent WGS. All the genes cited in the ROHHAD syndrome literature (see Introduction) were explored for coding region defects but no significant variants emerged. For this reason, an unbiased analysis for shared genetic etiology was performed. Overall, the mean depth of the six WGS for the two trios (#156 and #203) was 28.6X, ranging in the interval 27.5X-31.4X, with a mean of 77% (64.1%–86.3%) of the target genome covered at least 20X. In order to estimate whether coding regions were sufficiently covered, we specifically evaluated the sequencing depth in these regions and we observed >20X coverage in 95% and 96% of genes in proband #156 and #203, respectively. Therefore, while some limits may exist in detecting variants in intergenic regions, genes have coverage adequate for variant calling. The two probands were confirmed to be not related, and the parents of both families showed no consanguinity.

### Single nucleotide variants and small insertions/deletions

The total number of variants resulting from the Joint Genotyping Pipeline of DRAGEN for the WGS of the trios is summarized in Table 1.

**TABLE 1:** summary of the number of variants, divided by category, identified by the Dragen tools (single sample and trio analysis for the *de novo* variants), DNM2 and spliceAI tools.

		Rohhad #156			Rohhad #203		
		Affected	Mother	Father	Affected	Mother	Father
Dragen	total variants	4,910,681 (100%)	4,860,824 (100%)	4,875,654 (100%)	5,092,745 (100%)	5,071,664 (100%)	4,946,481 (100%)
	SNPs	4,102,454 (83.54%)	4,057,354 (83.47%)	4,064,660 (83.37%)	4,137,725 (81.25%)	4,125,598 (81.35%)	4,040,536 (81.69%)
	Indels (Het)	11,149 (0.23%)	11,010 (0.23%)	11,317 (0.23%)	20,857 (0.41%)	20,380 (0.4%)	17,545 (0.35%)
	Insertions (Hom)	158,994 (3.24%)	159,895 (3.29%)	152,922 (3.14%)	175,017 (3.44%)	177,160 (3.44%)	167,478 (3.39%)
	Insertions (Het)	227,803 (4.64%)	225,599 (4.64%)	237,066 (4.86%)	301,812 (5.93%)	313,369 (6.08%)	280,922 (5.68%)
	Deletions (Hom)	143,649 (2.93%)	143,486 (2.95%)	136,342 (2.80%)	148,859 (2.92%)	143,220 (2.78%)	149,622 (3.02%)
	Deletions (Het)	266,632 (5.43%)	263,480 (5.42%)	273,347 (5.61%)	308,475 (6.06%)	311,344 (6.04%)	290,378 (5.87%)
	*De novo* Autosome SNPs	320			544		
	*De novo* Autosome INDELs	257			347		
DNM2 tool	*De Novo* variant	70			86		
	Shared with Dragen	54			62		
SpliceAI	Splicing var (>0.5)	30			42		
	Splicing var (>0.5) *De Novo*	2			4		

Based on the sporadic occurrence of the disease in each family, the *de novo* variants were first taken into consideration. DRAGEN detected 320 and 544 *de novo* autosomal SNVs (DNM) and putative mendelian errors in proband #156 and #203, respectively. Given the number of *de novo* events estimated by DRAGEN far greater than expected, we also applied a second specific tool DenovoGear, which detected 70 and 86 DNM in the same patients. Of these, 54 variants are shared by the two tools in the proband #156 and 62 in the proband #203 (Supplementary Table S1). In particular, the variant annotation highlighted 37 of the 70 variants (52.9%) in proband #156 as “novel” versus 32 of the 86 (37.2%) in proband #203. In the former dataset, 42 variants overlapped transcripts, being mostly intronic (39% of total), intergenic (34% of total) and variants in regulatory regions (22%). Only two variants aroused interest: the already mentioned splicing variant in *SERPINE2* gene and a stop-gain variant in the *ZP4* gene. Similarly, in the #203 proband, 42 DNM overlapped transcripts with only one missense variant affecting the coding region of the *GPR75-ASB3* gene. All the estimated *de novo* mutations affecting genes have >20 × coverage (Supplementary Table S1, “mean coverage” column).

High impact variants of the coding region were then taken into consideration and compared between the two trios. As reported in Table 2 (details in Supplementary Table S2), no gene carrying pathogenic or likely pathogenic variants is shared between the two trios. Given the identification of a splicing variant in the *SERPINE2* gene, and the thorough targeted re-sequencing analysis already described in patient #156 and additional 22 ROHHAD patients, the whole gene locus was fully analyzed in the dataset of the #203 proband, both with visual inspection of the alignments and with bioinformatics tools, but no variant was identified.

**TABLE 2:** list of genes identified in the two trios, carrying variants with high impact (excluding synonymous and intronic) and genotype quality (GQ format field) ≥ 20.

#156 trio			#203 trio		
*de novo* ^a^	Compound heterozygous^b^	Recessive^c^	*de novo* ^a^	Compound heterozygous^b^	Recessive^c^
SERPINE2	CLDN23	AKR1C8P	ASB3	AKAP13	FCF1
ZP4	ERCC6	GPR107	SULT1C2	DOCK8	GAGE12G
	FRY	HLA-DQA1		GRIN3B	
	HLA-C			SULT1C2	
	HLA-DQA1			USP17L1	
	LIPE				
	PDILT				
	VPS8				

^a^ Criteria for *de novo* variants: Variants absent in parents (less than 2 reads allowed on parents alternate alleles), with frequency <0.001.

^b^ Criteria for compound-het: Variants with segregation analysis consistent with inheritance of each of the two variants from a different parent. For each variant, <10 individuals homozygous for the alternative allele must have been reported in the gnomAD database.

^c^ Criteria for recessive: Variants segregating according to recessive models in trios and a frequency across gnomAD population <0.01; Autosomal allele balance > 0.95.

### Splicing variants

Variants with a predicted impact on the splicing of a transcript were selected as reported in the method section. No homozygous or compound heterozygous splicing variant was called in either probands. Table 3 reports the *de novo* splicing variants in the two probands with the relative annotation. Although no pathogenic or likely pathogenic variant carrying gene is shared between the two families (Table 3; Supplementary Table S3), two splicing variants have been reported with a high impact prediction: *SERPINE2*, already known for #156, and *NCOA6* for #203. The *NCOA6* gene encodes a protein involved in the hormone-dependent transcriptional activation of nuclear receptors, including prostanoid, retinoid, vitamin D3, thyroid hormone, and steroid receptors.

**TABLE 3:** list of annotated *de novo* splicing variants in the two probands (spliceAI tool).

Sample	Chrom	POS	REF	ALT	Consequence	Impact	Gene
Rohhad #156	chr2	223998343	C	T	splice_acceptor_variant	High	SERPINE2
	chr2	227340384	A	G	splice_region_	Low	MFF
					variant &		
					intron_variant		
Rohhad #203	chr1	25834614	TCA	T	intron_variant	Modifier	AUNIP
	chr7	35245801	A	C	intron_variant	Modifier	TBX20
	chr20	34746930	T	A	splice_acceptor_variant	High	NCOA6
	chr21	45898905	A	G	intron_variant	Modifier	PCBP3

### Copy number variations (CNV)

*De novo* Copy Number Variations were selected based on the Dragen flag. In proband #156, a total of 51 *de novo* autosomal CNVs were selected and 17 of them overlapped 19 genes: 5 in the coding region (CDS) and 14 in untranslated regions (UTRs) or introns. One of these CNVs was already reported in DGV as benign while the others did not arouse any interest in relation to the present disease (Supplementary Table S4).

On the contrary, proband #203 appeared to have 174 *de novo* CNVs in autosomes, involving 278 genes in total. Most of them (64 of the 278) appeared entirely deleted, although this was not always confirmed by IGV inspections, while 93 of the remaining CNVs were found to affect coding regions. No pathogenic variation resulted from the analysis. After removing both variants reported as benign, variants associated with cancer (COSMIC annotation) or with unrelated diseases (OMIM annotation), 27 CNVs were retained (Supplementary Table S4). All were manually inspected by IGV and resulted to be false positives.

No common genes or overlapping regions between the two probands were identified. The recessive inheritance hypothesis was also tested but neither homozygous nor compound heterozygous variants involving the same transcripts could be identified in the two trios.

### Structural variants (SV)

Of the 13871 SVs detected by Dragen in the #156 trio, 266 high quality (PASS) variants were classified as *de novo*: 81 deletions, 4 duplications, 28 insertions, 10 inversions and 143 break ends (BND) calls. The latter are calls that can result from alignment artefacts or translocations, inversions, or more complex structural events. In the #203 trio, 32100 SVs were called, 661 of which were flagged as *de novo*. In particular, there were 167 deletions, 9 duplications, 222 insertions, 21 inversions and 242 BND calls. The comparison between genes involved in the *de novo* SVs of both probands revealed 27 common genes that, after investigation by IGV of alignment data, turned out to be false calls.

Variants classified, according to ACMG criteria, as pathogenic or likely pathogenic were also considered. Among 8 SVs thus selected in the #156 trio, only 2 presented a correct segregation in the family: a *de novo* duplication in chromosome 14 and a homozygous deletion in chromosome 4. Instead, the #203 trio had 13 SVs, two of which, located on chromosome 17, segregated correctly.

Based on the hypothesis of recessive inheritance, 363 and 503 SVs were selected in the #156 and #203 trios, respectively. After removal of 100 benign SVs in the first trio and 115 in the second, the remaining SVs were found to affect 121 genes in patient #156 (80 overlapping the CDS and 41 UTRs) and 115 genes in patient #203 (135 overlapping the CDS and 48 UTRs). The genes *TPO, WT1, GPC5* and *TAFA5* were shared between the two lists. However, they were discarded as unrelated to any ROHHAD symptom, and their corresponding variants affected only intronic regions.

Finally, given the intriguing similarities between ROHHAD and Prader-Willi Syndrome (PWS), although considered of limited interest (Barclay et al., 2018), a careful analysis of both *de novo* and inherited SVs was carried out regarding the proximal portion of the long arm of chromosome 15, without finding any variant shared between the two probands able to explain their phenotype.

### Tandem repeats

Alleles comprising a number of repeated units different than expected were surveyed for genes already known to cause diseases with a mechanism of expansion of Simple Tandem Repeats (STR), without finding any shared evidence able to account for the ROHHAD phenotype of the two probands.

## Discussion

ROHHAD syndrome is a yet unsolved diagnostic challenge. Although all affected patients show the same symptoms (Rapid-onset Obesity with Hypothalamic dysfunction, Hypoventilation and Autonomic Dysregulation with possible tumors of the sympathetic nervous system), these can occur with variable timing between the disease onset—usually with hyperphagic obesity as the first disease manifestation—and the appearance of further symptoms, without a precise order and with unique peculiarities (Gharial et al., 2021; Ozcan et al., 2022). Being highly disabling and often fatal, ROHHAD requires rapid identification and timely treatment of clinical manifestations, especially sleep-disordered-breathing, sodium imbalance and hormonal defects. Furthermore, since the etiopathogenesis is still unknown, so far it has only been possible to establish guidelines and suggestions for rapid disease recognition and consequent management (Harvengt et al., 2019; Filippidou et al., 2020; Selvadurai et al., 2020; Amjadipour et al., 2021; Basiratnia et al., 2021).

Twenty-three ROHHAD patients have so far been recruited in the Pediatric Endocrinology Unit of the Gaslini Children’s Hospital in Genoa (Italy). In search of a possible shared genetic cause, two of these probands and their parents were selected—due to their severe, complete ROHHAD phenotype—to undergo WGS.

Different types of variants were considered, either *de novo* or transmitted through any possible pattern of inheritance, however none of the variants thus identified is likely to be pathogenic and affect a gene having function and expression pattern consistent with the ROHHAD clinical phenotype, therefore they were not validated by Sanger sequencing. Furthermore, we focused on common variants between the two ROHHAD patients, thus filtering out also, but not limited to, false positives for both SNV and CNV/SV. Nonetheless, in the absence of an experimental validation, impracticable given the very high number of variants detected, no variant can be considered a true positive.

Regarding the SNVs, Dragen identified a greater number of *de novo* events than DeNovoGear. When focusing on variants detected by both software (Table 1), which are the variants more likely to be valid, none of them was present in both patients.

Both probands underwent genome-wide CGH array, which resulted negative. Since these tests were performed in a diagnostic setting, only CNVs already known to be pathogenic and encompassing more than three probes were reported. Unfortunately, this prevented us from using the whole set of CNVs detected by aCGH to estimate the false positive and false negative rates of the CNVs identified by sequencing.

Based on our results and other similar findings (Barclay et al., 2022), the argument for a Mendelian genetic cause for ROHHAD is slowly losing value, while new hypotheses seem more promising, like the epigenetic theory, the possibility of a paraneoplastic syndrome, an autoimmune syndrome and, finally, the need for a trigger, such as the case in which the SARS-CoV-2 virus infection may have induced secondary symptoms of ROHHAD (Cemeroglu et al., 2016; Lazea et al., 2021; Artamonova et al., 2022; Tiwari et al., 2022). Up to now, only Artamonova et al. (2022) have described the onset of ROHHAD after a viral infection, however, as no increase in the incidence of ROHHAD has apparently been reported since the start of the COVID-19 pandemic, this circumstance needs to be verified over time. The hypothesis of a genetic predisposition that requires an environmental trigger (possibly an immune factor such as that which occurs during an infectious event) for the disease to manifest—as seen in other autoimmune conditions - could explain the fact that ROHHAD patients generally show no symptoms in the first 2–4 years of their life. This is certainly engaging and deserves further investigation, keeping in mind, however, that the presence of a neural tumor in up to 40% of patients appears to be inconsistent with this “infection trigger” hypothesis.

The recent identification of a specific autoantigen in ROHHAD patients with neural tumors and “active” disease—with positive ZSCAN1 autoantibodies both in peripheral blood and CSF, and ZSCAN1 expression in neural tumor tissues—supports the autoimmune-paraneoplastic hypothesis (Mandel-Brehm et al., 2022). The autoimmune hypothesis is supported also by other findings, such as CSF oligoclonal bands (Sartori et al., 2014), blood and CSF anti hypothalamus and anti-pituitary autoantibodies (Giacomozzi et al., 2019), and response to immunosuppressive or immune-modulating therapy (Jacobson et al., 2016; Hawton et al., 2022).

Interestingly, based on previous reports on the overlap between ROHHAD and other hypothalamic conditions, the author of a recent review has hypothesized a common aetiology for ROHHAD syndrome and autoimmune adipsic hypernatremia, in which specific autoantibodies against subfornical organ and/or anti-sodium sensor have been detected (Nakamura-Utsunomiya, 2022). Clinically, the two syndromes differ in that ROHHAD patients’ hypernatremia is often transient and adipsia is not always observed (our case-series, data not shown). To date, however, no single explanation seems to be fully exhaustive, though all together they can contribute to better clarify the clinical picture of ROHHAD patients.

We are also aware that our results may be affected by several factors such as the use of short-read sequencing that strongly influences our capability to identify variants other than SNPs and Indels, low-coverage in noncoding regions, accuracy of the software for variant detection and selected thresholds, and therefore we cannot rule out that pathogenic variants could not be found due to low sensitivity. On the other hand the approaches used in our work are widespread for the study of germline mutations in WGS.

Continuous technological development can help solve this intriguing “ROHHAD” challenge. Indeed, to discover the pathogenic mechanism(s) underlying ROHHAD we need to further explore the WGS datasets and take advantage of third-generation sequencing technologies (e.g., long-read sequencing, optical mapping, etc.). Furthermore, new pathways will have to be investigated, including those associated with immunological and autoimmune disorders, making use not only of genomics but also of different -omic datasets.

## Data Availability

The original contributions presented in the study are included in the article/Supplementary Material, further inquiries can be directed to the corresponding author.
