# Supplementary material for: Whole genome sequencing in ROHHAD trios proved inconclusive: what’s beyond?
Source: Front Genet. 2023 Aug 7;14:1031074. doi: 10.3389/fgene.2023.1031074 (PMC10440434; doi:10.3389/fgene.2023.1031074)
Supplement: Supplementary file 3 [file Presentation1.PPTX]

## Slide 1
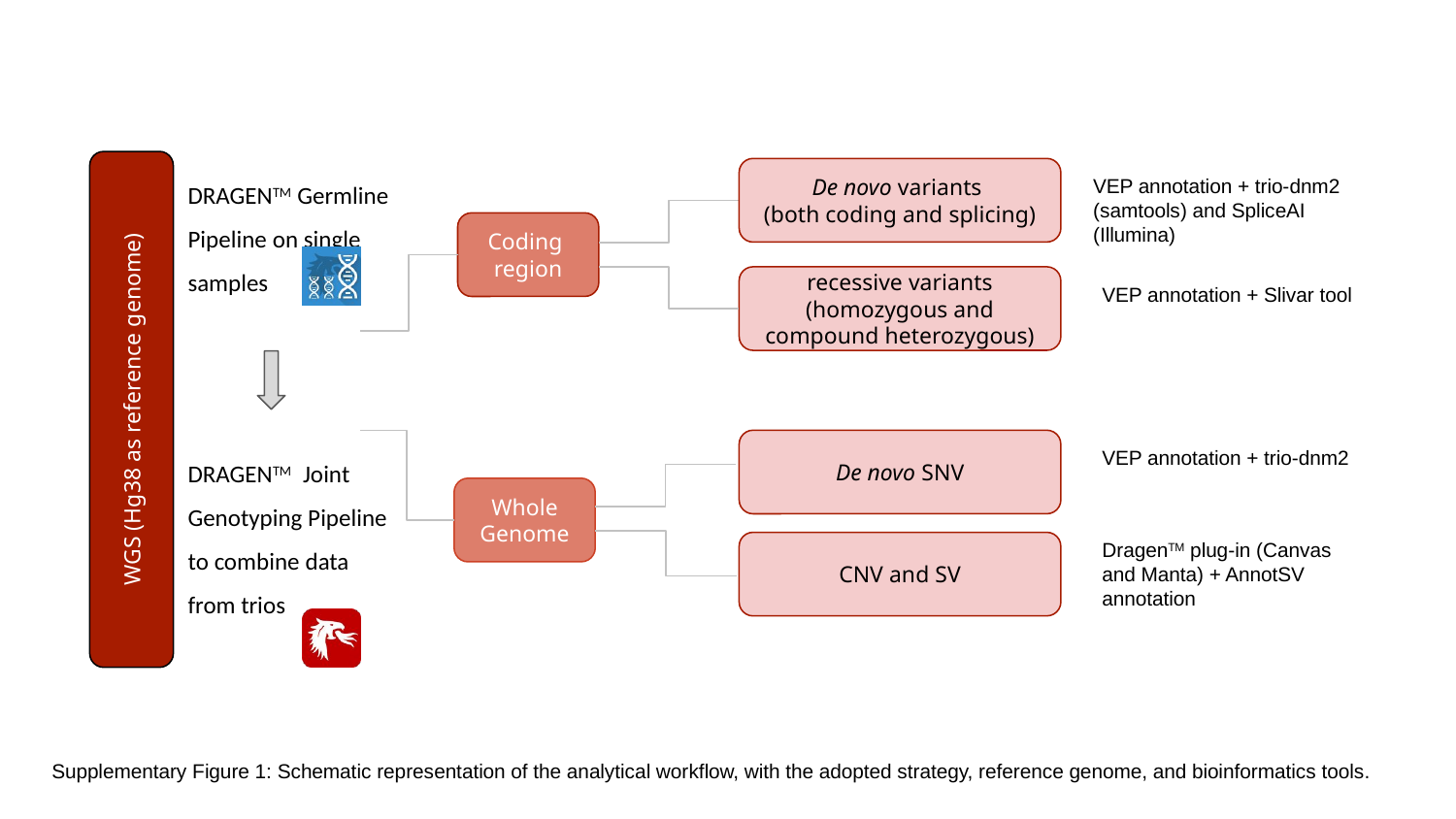

DRAGENTM Germline Pipeline on single samples
DRAGENTM Joint Genotyping Pipeline to combine data from trios
De novo variants
(both coding and splicing)
VEP annotation + trio-dnm2 (samtools) and SpliceAI (Illumina)
Coding
region
recessive variants (homozygous and compound heterozygous)
VEP annotation + Slivar tool
WGS (Hg38 as reference genome)
De novo SNV
VEP annotation + trio-dnm2
Whole Genome
DragenTM plug-in (Canvas and Manta) + AnnotSV annotation
CNV and SV
Supplementary Figure 1: Schematic representation of the analytical workflow, with the adopted strategy, reference genome, and bioinformatics tools.
