# Supplementary material for: Whole genome sequencing in ROHHAD trios proved inconclusive: what’s beyond?
Source: Front Genet. 2023 Aug 7;14:1031074. doi: 10.3389/fgene.2023.1031074 (PMC10440434; doi:10.3389/fgene.2023.1031074)
Supplement: Supplementary file 6 [file DataSheet1.DOCX]

Supplement 1:

This supplement provides the criteria that were used in order to diagnose the different clinical manifestations of ROHHAD syndrome in our patients.

HYPERPROLACTINEMIA: hyperprolactinemia was defined as prolactin levels higher than normal reference range, that failed to decrease 30 and 60 minutes after the positioning of an indwelling venous cannula, so to remove the “emotional stress” factor (Melmed et al., 2011). Idiopathic hyperprolactinemia was confirmed after ruling out all causes of secondary hyperprolactinemia, such as pituitary adenoma, hypothalamic-pituitary lesions or pituitary stalk damage, primary hypothyroidism, kidney failure, pregnancy or medications that are known to cause an increase in prolactin levels.

GROWTH HORMONE DEFICIENCY: GH deficiency was diagnosed in both patients according to current consensus guidelines at time of diagnosis (Growth Hormone Research Society, 2000), based on clinical criteria, IGF-I levels and response to two different GH stimulation tests performed on two separate days.

CENTRAL ADRENAL INSUFFICIENCY: Central adrenal insufficiency was suspected when patient showed symptoms such as fatigue, nausea, poor response to illness or stress, and basal cortisol levels were below 3 mg/dl (83 nmol/L), and/or cortisol response to ITT or low-dose ACTH test was insufficient (Patti et al., 2018).

CENTRAL HYPOTHYROIDISM: central hypothyroidism was diagnosed based on low serum levels of FT4 – below reference range provided by laboratory assay – confirmed on at least two samples taken on two different days, with patient in basal conditions and not during severe illnesses, with normal, low or only slightly elevated TSH levels (Krude et al., 2011).

CENTRAL PRECOCIOUS PUBERTY: the appearance of thelarche before age 8 years, associated with an increase in growth velocity, advanced bone age, ultrasound evidence of pubertal internal genitalia, elevated blood levels of estrogens and LH, with pubertal increase of LH and FSH after GnRH stimulation test (Léger et al., 2015).

CENTRAL HYPOGONADISM: in females, lack of breast development by 13 years, a delay of over 4 years between thelarche and completion of puberty, are clinical signs of delayed puberty and suspected central hypogonadism, which can be confirmed by low basal levels of FSH, LH and estrogen levels, and confirmed by GnRH test (Boehm et al., 2015).

CENTRAL DIABETES INSIPIDUS: polyuria and polydipsia in association with elevated plasma sodium and plasma osmolality levels (basal or after water deprivation test) and with low urine osmolality suggest diabetes insipidus. Response to nasal or intramuscular DDAVP – with an increase in urine osmolality and a decrease in plasma osmolality – differentiates central from nephrogenic forms. Central diabetes insipidus may be complete or partial. (Patti et al., 2022).

CENTRAL HYPOVENTILATION: Central hypoventilation is suspected when CO2 is elevated during wake and/or sleep time, in the absence of lung disease or neuromuscular disease that may compromise ventilation or alveolar gas exchange. Elevated CO2 (>50mmHg) – measured by means of arterial gas analysis, transcutaneous or end-tidal sensors - for >25% of the total sleep time allows the diagnosis of sleep hypoventilation (Berry et al., 2012).

CENTRAL APNEAS: In children >1 year of age, a respiratory event (≥ 90% drop of oronasal flow or alternative apnea sensor signal) can be defined as a central apnea if it is associated with absent inspiratory effort throughout the entire duration of the event, and at least one of the following is met:

1. The event lasts 20 seconds or longer.

2. The event lasts at least the duration of two breaths during baseline breathing and is associated with an arousal or ≥3% oxygen desaturation (Berry et al., 2012).

THERMAL DYSREGULATION: we defined thermal dysregulation as the appearance of frequent episodes of fever without signs of infections, or episodes when body temperature dropped below 35°C – hypothermia –; both high and low body temperature were accompanied by excessive sweating in our ROHHADNET patients. No specific test for thermal stability was used in these patients.

BRADYCARDIA: bradycardia, both during sleep and during wake, was defined as low heart rate for age that persisted for 15 seconds or more, according to published tables (Fleming et al., 2011). When bradycardia was detected during patient exam or overnight monitoring, a 24 hours ECG monitoring was performed to confirm this finding.

References:

Berry RB, Budhiraja R, Gottlieb DJ, Gozal D, Iber C, Kapur VK, Marcus CL, Mehra R, Parthasarathy S, Quan SF, Redline S, Strohl KP, Davidson Ward SL, Tangredi MM; American Academy of Sleep Medicine. Rules for scoring respiratory events in sleep: update of the 2007 AASM Manual for the Scoring of Sleep and Associated Events. Deliberations of the Sleep Apnea Definitions Task Force of the American Academy of Sleep Medicine. J Clin Sleep Med. 2012 Oct 15;8(5):597-619

Boehm U, Bouloux PM, Dattani MT, de Roux N, Dodé C, Dunkel L, Dwyer AA, Giacobini P, Hardelin JP, Juul A, Maghnie M, Pitteloud N, Prevot V, Raivio T, Tena-Sempere M, Quinton R, Young J. Expert consensus document: European Consensus Statement on congenital hypogonadotropic hypogonadism--pathogenesis, diagnosis and treatment. Nat Rev Endocrinol. 2015 Sep;11(9):547-64

Fleming S, Thompson M, Stevens R, Heneghan C, Plüddemann A, Maconochie I, Tarassenko L, Mant D. Normal ranges of heart rate and respiratory rate in children from birth to 18 years of age: a systematic review of observational studies. Lancet. 2011 Mar 19;377(9770):1011-8

Growth Hormone Research Society. Consensus guidelines for the diagnosis and treatment of growth hormone (GH) deficiency in childhood and adolescence: summary statement of the GH Research Society. GH Research Society. J Clin Endocrinol Metab. 2000 Nov;85(11):3990-3

Krude H, Grüters A: Diagnostic tests of thyroid function in children and adolescents. In: Ranke MB, Mullis PE (eds): Diagnostics of Endocrine Function in Children and Adolescents, ed. 4, Basel, Karger, 2011, pp 85-101

Léger J, Carel JC. Central Precocious Puberty - Management and Long-term Outcomes. Eur Endocrinol. 2015 Apr;11(1):45-46. doi: 10.17925

Melmed S, Casanueva FF, Hoffman AR, Kleinberg DL, Montori VM, Schlechte JA, et al; Endocrine Society. Diagnosis and treatment of hyperprolactinemia: an Endocrine Society clinical practice guideline. J Clin Endocrinol Metab 2011;96:273-88. doi:10.1210/jc.2010- 1692

Patti G, Guzzeti C, Di Iorgi N, Maria Allegri AE, Napoli F, Loche S, Maghnie M. Central adrenal insufficiency in children and adolescents. Best Pract Res Clin Endocrinol Metab. 2018 Aug;32(4):425-444. doi: 10.1016

Patti G, Napoli F, Fava D, Casalini E, Di Iorgi N, Maghnie M. Approach to the Pediatric Patient: Central Diabetes Insipidus. J Clin Endocrinol Metab. 2022 Apr 19;107(5):1407-1416
